# The prognostic impact of BMI in patients with HR+/HER2− advanced breast cancer: a study of the SONABRE registry

**DOI:** 10.1007/s10549-023-07108-6

**Published:** 2023-10-25

**Authors:** Senna W. M. Lammers, Hannah Thurisch, Ingeborg J. H. Vriens, Marissa Meegdes, Sanne M. E. Engelen, Frans L. G. Erdkamp, M. Wouter Dercksen, Birgit E. P. J. Vriens, Kirsten N. A. Aaldering, Manon J. A. E. Pepels, Linda M. H. van de Winkel, Natascha A. J. B. Peters, Jolien Tol, Joan B. Heijns, Agnes J. van de Wouw, Nathalie J. A. Teeuwen, Sandra M. E. Geurts, Vivianne C. G. Tjan-Heijnen

**Affiliations:** 1 Department of Medical Oncology, Maastricht University Medical Centre, GROW, Maastricht University, P.O. Box 5800, 6202 AZ Maastricht, The Netherlands; 2 Department of Surgery, Maastricht University Medical Centre, Maastricht, The Netherlands; 3 Department of Internal Medicine, Zuyderland Medical Centre, Sittard-Geleen, The Netherlands; 4 Department of Medical Oncology, Máxima Medical Centre, Eindhoven, The Netherlands; 5 Department of Internal Medicine, Catharina Hospital, Eindhoven, The Netherlands; 6 Department of Internal Medicine, Laurentius Hospital, Roermond, The Netherlands; 7 Department of Internal Medicine, Elkerliek Hospital, Helmond, The Netherlands; 8 Department of Internal Medicine, St. Anna Hospital, Geldrop, The Netherlands; 9 Department of Internal Medicine, St. Jans Gasthuis, Weert, The Netherlands; 10 Department of Internal Medicine, Jeroen Bosch Hospital, Den Bosch, The Netherlands; 11 Department of Internal Medicine, Amphia Hospital, Breda, The Netherlands; 12 Department of Internal Medicine, Viecuri Medical Centre, Venlo, The Netherlands

**Keywords:** Metastatic breast cancer, Hormone receptor-positive, Body mass index, Overall survival, Progression-free survival, Endocrine therapy

## Abstract

**Purpose:**

This study determines the prognostic impact of body mass index (BMI) in patients with hormone receptor-positive/human epidermal growth factor receptor-2-negative (HR+/HER2−) advanced (i.e., metastatic) breast cancer (ABC).

**Methods:**

All patients with HR+/HER2− ABC who received endocrine therapy +—a cyclin-dependent kinase 4/6 inhibitor as first-given systemic therapy in 2007–2020 in the Netherlands were identified from the Southeast Netherlands Advanced Breast Cancer (SONABRE) registry (NCT03577197). Patients were categorised as underweight (BMI: < 18.5 kg/m^2^), normal weight (18.5–24.9 kg/m^2^), overweight (25.0–29.9 kg/m^2^), or obese (≥ 30.0 kg/m^2^). Overall survival (OS) and progression-free survival (PFS) were compared between BMI classes using multivariable Cox regression analyses.

**Results:**

This study included 1456 patients, of whom 35 (2%) were underweight, 580 (40%) normal weight, 479 (33%) overweight, and 362 (25%) obese. No differences in OS were observed between normal weight patients and respectively overweight (HR 0.99; 95% CI 0.85–1.16; p = 0.93) and obese patients (HR 1.04; 95% CI 0.88–1.24; p = 0.62). However, the OS of underweight patients (HR 1.45; 95% CI 0.97–2.15; p = 0.07) tended to be worse than the OS of normal weight patients. When compared with normal weight patients, the PFS was similar in underweight (HR 1.05; 95% CI 0.73–1.51; p = 0.81), overweight (HR 0.90; 95% CI 0.79–1.03; p = 0.14), and obese patients (HR 0.88; 95% CI 0.76–1.02; p = 0.10).

**Conclusion:**

In this study among 1456 patients with HR+/HER2− ABC, overweight and obesity were prevalent, whereas underweight was uncommon. When compared with normal weight, overweight and obesity were not associated with either OS or PFS. However, underweight seemed to be an adverse prognostic factor for OS.

**Supplementary Information:**

The online version contains supplementary material available at 10.1007/s10549-023-07108-6.

## Introduction

The prevalence of obesity has been rising for decades [1]. It is estimated that over one fifth of the female world population will be obese by the year 2025 [1]. Obesity is a well-known risk factor for the development of breast cancer in postmenopausal women and has been associated with a higher risk of disease recurrence and death following a diagnosis of early breast cancer (EBC) [2–6]. The causal relationship between obesity and breast cancer risk and prognosis is complex, but might, at least in part, be explained by an increased peripheral conversion of androgens to oestrogens [7].

A number of patients with breast cancer will eventually develop (distant) metastases [8]. In advanced (i.e., metastatic) breast cancer (ABC), mixed results have been reported on the prognostic effect of overweight and obesity [9–19]. Interpretation of these results is furthermore complicated by differences in patient and treatment characteristics, study population size, body mass index (BMI) categorisation, and endpoints. Moreover, the majority of studies in ABC exclude underweight patients or do not categorise them as a separate group of patients [10, 11, 14–19]. The French ESME cohort study however recently showed that underweight patients with ABC have a lower overall survival (OS) and first-line progression-free survival (PFS) when compared with normal weight patients with ABC [12].

Apart from clarifying the prognostic effect of BMI in patients with ABC, it might also be important to study potential effect modifiers, such as patient, tumour, and treatment characteristics. In the general population, for example, it was shown that the association between BMI and all-cause-mortality tends to differ by age [20]. In fact, several studies observed that overweight, when compared with normal weight, was a protective factor for all-cause mortality in older adults [21–23]. In patients with EBC, age-dependent associations between BMI and death from any cause have also been reported (Lammers S.W.M., Geurts S.M.E., van Hellemond I.E.G. et al. *The prognostic and predictive effect of body mass index in hormone receptor-positive breast cancer* [submitted for publication]) [24]. In addition, following advancements in systemic therapy over the years (i.e., the introduction of cyclin-dependent kinase (CDK) 4/6 inhibitors in the treatment of patients with hormone receptor-positive/human epidermal growth factor receptor-2-negative (HR+/HER2−) ABC), the prognostic effect of BMI might be modified by period and type of treatment [25].

The current study therefore aimed to address two research questions in a real-world cohort of patients diagnosed with HR+/HER2− ABC between 2007 and 2020 in the Netherlands. All patients received endocrine therapy with or without a CDK 4/6 inhibitor as first-given systemic therapy. The primary aim of this study was to evaluate whether BMI is an independent prognostic factor for both OS and PFS. The secondary aim of this study was to evaluate whether this prognostic effect of BMI is modified by age at diagnosis, period of treatment, or type of treatment.

## Methods

### Study design and population

Patients were identified from the Southeast Netherlands Advanced Breast Cancer (SONABRE) registry (NCT03577197), an ongoing prospectively maintained retrospective cohort study [26]. The SONABRE registry includes all patients (≥ 18 years) diagnosed with de novo or recurrent ABC from eleven hospitals in the southeast of the Netherlands since 2007. Information about patient, tumour, and treatment characteristics is retrospectively collected from medical files by trained registration clerks. Treatment, progression per treatment line, and survival data are updated annually.

For the current analysis, all patients diagnosed with HR+/HER2− ABC who received endocrine therapy with or without a CDK 4/6 inhibitor as first-given systemic therapy between 2007 and 2020 were identified from ten participating hospitals. Of note, in the Netherlands, CDK 4/6 inhibitors were implemented for treatment of HR+/HER2− ABC in August 2017 [27]. Patients with an unknown BMI at diagnosis were excluded as well as patients who received another type of systemic therapy or no systemic therapy. Data lock was on November 11, 2022.

Approval for the SONABRE registry was obtained from the Medical Research Ethics Committee of the Maastricht University Medical Centre (15-4-239).

### Definitions

Tumours were considered HR+ if ≥ 10% of invasive cells had a positive nuclear staining of oestrogen and/or progesterone receptors. HER2-negativity was defined by an immunohistochemistry score of 0 or 1 or a negative fluorescence in situ hybridization result.

BMI was calculated from weight and height (BMI = weight [kg]/height [m]^2^), measured by the treating physician or self-reported by the patient at diagnosis. In accordance with the World Health Organization criteria, BMI was categorised as underweight (< 18.5 kg/m^2^), normal weight (18.5–24.9 kg/m^2^), overweight (25.0–29.9 kg/m^2^), or obese ($\ge$ 30.0 kg/m^2^).

Metastatic-free interval (MFI) reflects the time between primary breast cancer diagnosis and diagnosis of metastatic disease. An MFI of < 3 months was considered de novo metastatic disease. Endocrine resistance was defined as experiencing a relapse during or within 12 months after finishing adjuvant endocrine therapy. Endocrine sensitivity was defined as experiencing a relapse more than 12 months after completing adjuvant endocrine therapy or having no prior exposure to endocrine therapy.

### Endpoints

The primary endpoint was OS, defined as the time between the start of first-given systemic therapy for ABC and the date of death from any cause. The secondary endpoint was PFS, defined as the time between the start of first-given systemic therapy for ABC and the date of progression or death. Progression was defined as occurrence of a new metastatic site or progression of existing metastases. These findings were based on imaging, the presence of tumour markers, and/or the presence of symptoms.

### Statistical analysis

Baseline characteristics were compared between BMI classes using the Chi-squared test (categorical variables) and the Kruskal–Wallis test (continuous variables).

Median OS and PFS were calculated using the Kaplan–Meier method. Differences between BMI classes were assessed with the log-rank test. In the absence of an event, patients were censored at the last follow-up date. Patients subjected to a new line of therapy due to toxicity without progression of disease were also censored in the analysis of PFS as of the date of start of new treatment.

Multivariable Cox proportional hazards regression analyses were performed to evaluate whether BMI remained an independent prognostic factor for both OS and PFS. Multivariable analyses were performed in the total study population and in patients with metachronous metastases. Prognostic factors with a univariable p-value of ≤ 0.10 were included in the multivariable analyses. The following potential confounding factors were considered: age, WHO performance status, presence of comorbidities, MFI, number of metastatic sites, and site of metastases [12, 25]. In patients with metachronous metastases, endocrine sensitivity was included as an additional confounding factor.

As the association between BMI and all-cause mortality differs by age in the general population [20] and systemic treatment of patients with HR+/HER2− ABC changed over time [25], analyses were stratified by age at diagnosis of ABC (< 60 versus ≥ 60 years), period of treatment (2007–2011 versus 2012–2016 versus 2017–2021), and type of first-line treatment (endocrine monotherapy versus endocrine therapy with a CDK 4/6-inhibitor). The BMI-by-age, BMI-by-period, and BMI-by-treatment interaction terms were calculated using likelihood ratio tests.

All statistical tests were conducted two-sided with a statistical significance threshold of p ≤ 0.05 and performed with SPSS (version 25) and Stata (version 17).

## Results

### Patient characteristics

**Fig. 1:**
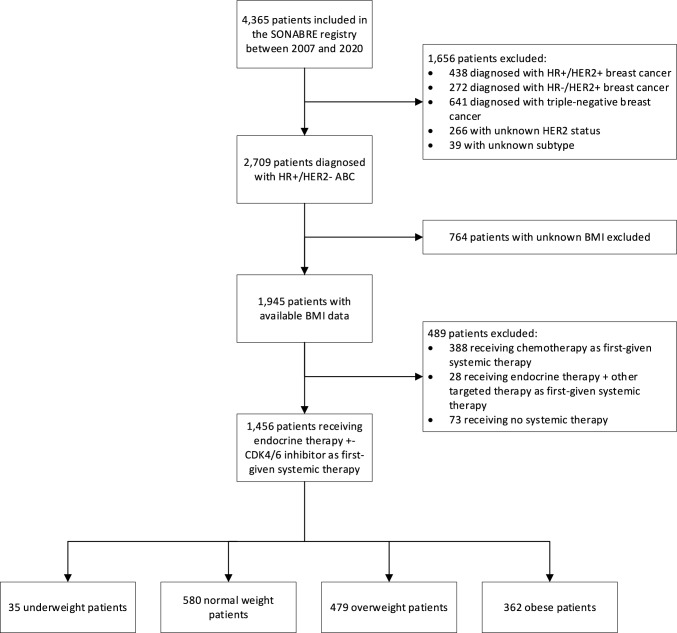
Flowchart of included patients

Of 4365 patients included in the SONABRE registry between 2007 and 2020, 2709 patients were diagnosed with HR+/HER2− ABC (Fig. 1). After exclusion of patients without a BMI measurement at diagnosis (n = 764) or patients who did not receive endocrine therapy with or without a CDK 4/6 inhibitor as first-given systemic therapy (n = 489), the eligible study population consisted of 1456 patients. Among these patients were 35 (2%) underweight, 580 (40%) normal weight, 479 (33%) overweight, and 362 (25%) obese patients.

The presence of comorbidities and bone-only metastases increased significantly with an increasing BMI class, whereas the presence of visceral metastases decreased (p ≤ 0.001) (Table 1). When compared with other BMI classes, underweight patients had a worse WHO performance status and were more frequently diagnosed with de novo metastatic disease (p ≤ 0.001). In patients with metachronous metastases, the presence of endocrine sensitivity slightly differed between BMI classes (p = 0.04) (Supplementary Table 1). When compared with the percentage of endocrine-resistant patients (28%) in normal weight patients, the percentage of endocrine-resistant patients was higher in underweight (35%), overweight (35%), and obese patients (38%).

**Table 1:** Baseline characteristics of the study population, overall and according to BMI class at diagnosis of HR+/HER2− ABC (N [%])

	Total n = 1456 (100%)	Underweight n = 35 (2%)	Normal weight n = 580 (40%)	Overweight n = 479 (33%)	Obese n = 362 (25%)	p-value
Sex						0.58
Female	1439 (99)	35 (100)	575 (99)	471 (98)	358 (99)	
Median age—years (range)	68 (29–98)	68 (47–91)	68 (29–98)	69 (36–93)	68 (33–89)	0.37
Age						0.59
< 60 years	398 (27)	9 (26)	170 (29)	125 (26)	94 (26)	
≥ 60 years	1058 (73)	26 (74)	410 (71)	354 (74)	268 (74)	
WHO performance status						< 0.001
0–1	1103 (83)	14 (52)	429 (82)	388 (88)	272 (81)	
2–4	227 (17)	13 (48)	96 (18)	55 (12)	63 (19)	
Unknown	126	8	55	36	27	
Comorbidities^a^						< 0.001
No	521 (36)	13 (37)	252 (43)	158 (33)	98 (27)	
Yes	935 (64)	22 (63)	328 (57)	321 (67)	264 (73)	
MFI						< 0.001
< 3 months (de novo)	372 (26)	15 (43)	120 (21)	120 (25)	117 (32)	
3–60 months (recurrent)	354 (24)	5 (14)	138 (24)	118 (25)	93 (26)	
> 60 months (recurrent)	730 (50)	15 (43)	322 (56)	241 (50)	152 (42)	
Number of metastatic sites						0.33
Single	691 (48)	17 (49)	259 (45)	232 (48)	183 (51)	
Multiple	765 (53)	18 (51)	321 (55)	247 (52)	179 (49)	
Site of metastases						0.04
Bone only	507 (35)	11 (31)	178 (31)	177 (37)	141 (39)	
Soft tissue, without visceral or CNS manifestations	184 (13)	2 (6)	66 (11)	64 (13)	52 (14)	
Visceral or other, without CNS manifestations	725 (50)	22 (63)	318 (55)	223 (47)	162 (45)	
CNS	40 (3)	0 (0)	18 (3)	15 (3)	7 (2)	
(Neo)adjuvant treatment						0.12
No (incl. MFI < 3 months)	665 (46)	20 (57)	247 (43)	220 (46)	178 (49)	
Yes	791 (54)	15 (43)	333 (57)	259 (54)	184 (51)	
(Neo)adjuvant endocrine treatment						0.40
No (incl. MFI < 3 months)	710 (49)	20 (57)	270 (47)	235 (49)	185 (51)	
Yes	746 (51)	15 (43)	310 (53)	244 (51)	177 (49)	
Endocrine sensitivity						0.40
Resistant	354 (24)	7 (20)	129 (22)	126 (26)	92 (25)	
Sensitive	1102 (76)	28 (80)	451 (78)	353 (74)	270 (75)	
Type of first-line endocrine therapy for ABC						0.39
Aromatase inhibitor	1161 (80)	31 (89)	452 (78)	392 (82)	286 (79)	
Tamoxifen	165 (11)	4 (11)	75 (13)	48 (10)	38 (11)	
Fulvestrant	121 (8)	0 (0)	49 (8)	38 (8)	34 (9)	
Other	9 (1)	0 (0)	4 (1)	1 (< 1)	4 (1)	

Data in each cell represent numbers with associated percentages, unless otherwise specified

Percentages may exceed 100% due to rounding

*ABC *advanced breast cancer, *CNS *central nervous system, *HR+/HER2−* hormone receptor-positive/human epidermal growth factor receptor-2-negative, *MFI *metastatic-free interval, *WHO *World Health Organization

^a^The definition of comorbidities includes the following conditions: diabetes mellitus, cerebrovascular disease, connective tissue disease, dementia, hypertension, chronic pulmonary disease, liver disease, renal disease, AIDS, peptic ulcer, leukaemia, malignant lymphoma, myocardial infarction, malignant solid tumour (excluding breast cancer and basal cell carcinoma of the skin), osteoporosis, chronic bowel disease, chronic infectious disease, atherosclerosis, heart failure, mental health conditions, thromboembolism, and heart rhythm disorders

Overall, 1200 patients received endocrine monotherapy and 256 patients received endocrine therapy in combination with a CDK 4/6 inhibitor as first-given systemic therapy between 2007 and 2020. After the implementation of CDK 4/6 inhibitors in the Netherlands, between 2017 and 2020, 31% of patients received endocrine therapy in combination with a CDK 4/6 inhibitor as first-given systemic therapy (Supplementary Figure 1). The use of CDK 4/6 inhibitors was similar between BMI classes (p = 0.87). In the total study population (n = 1456), the majority of patients (80%) received an aromatase inhibitor as first-line endocrine therapy (Table 1). All other patients received either tamoxifen (11%), fulvestrant (8%), or another type of endocrine therapy (1%). First-line endocrine therapy choices were equally distributed among BMI classes (p = 0.39).

### Prognostic impact of BMI on OS

**Fig. 2:**
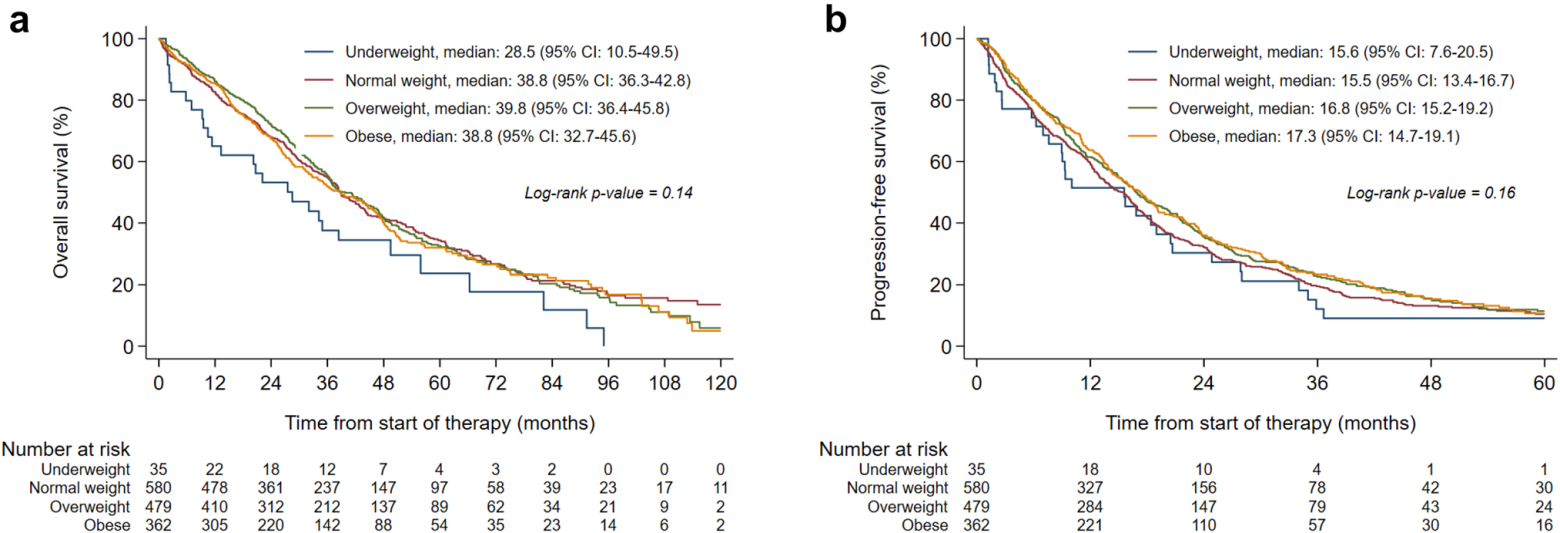
Overall survival (**a**) and first-line progression-free survival (**b**) according to BMI class at diagnosis of HR+/HER2− ABC

The median follow-up time of the total study population was 60.9 months (IQR 37.5–96.0). No statistically significant difference in OS was observed between BMI classes, with a median OS of 28.5 months (95% confidence interval (CI) 10.5–49.5) in underweight, 38.8 months (95% CI 36.3–42.8) in normal weight, 39.8 months (95% CI 36.4–45.8) in overweight, and 38.8 months (95% CI 32.7–45.6) in obese patients (log-rank p-value = 0.14) (Fig. 2a). However, after adjustment for potential confounders, the OS of underweight patients tended to be worse than the OS of normal weight patients (hazard ratio (HR) 1.45; 95% CI 0.97–2.15; p = 0.07), though not statistically significant. The OS of overweight and obese patients was similar to the OS of normal weight patients (adjusted HR 0.99; 95% CI 0.85–1.16; p = 0.93 and adjusted HR 1.04; 95% CI 0.88–1.24; p = 0.62, respectively) (Table 2). In patients with metachronous metastases, the detrimental effect of underweight on OS was stronger and statistically significant (adjusted HR 1.85; 95% CI 1.13–3.05; p = 0.02) (Supplementary Figure 2a and Supplementary Table 2; model 3). The prognostic effect of BMI on OS was not modified by age at diagnosis, period of treatment, or type of first-line treatment (Table 2).

**Table 2:** Multivariable analyses of overall survival according to BMI class at diagnosis of HR+/HER2− ABC in the total study population and subgroups of patients

	BMI							p interaction
	Underweight		Normal weight	Overweight		Obese		
	HR (95% CI)	p-value	Reference	HR (95% CI)	p-value	HR (95% CI)	p-value	
Overall survival								
All patients (n = 1456 patients, 943 events)^a^	1.45 (0.97–2.15)	0.07	1.00	0.99 (0.85–1.16)	0.93	1.04 (0.88–1.24)	0.62	
Age at diagnosis of ABC								0.96
< 60 years (n = 398 patients, 243 events)	1.28 (0.58–2.84)	0.54	1.00	1.01 (0.75–1.36)	0.96	0.98 (0.70–1.38)	0.92	
≥ 60 years (n = 1058 patients, 700 events)	1.29 (0.81–2.04)	0.28	1.00	1.01 (0.85–1.21)	0.91	1.05 (0.86–1.27)	0.66	
Period of treatment								0.31
2007–2011 (n = 179 patients, 168 events)	1.04 (0.43–2.51)	0.92	1.00	0.94 (0.63–1.40)	0.75	1.33 (0.88–2.00)	0.17	
2012–2016 (n = 518 patients, 411 events)	2.43 (1.30–4.56)	0.005	1.00	0.91 (0.73–1.15)	0.45	0.95 (0.72–1.24)	0.69	
2017–2021 (n = 759 patients, 364 events)	1.37 (0.70–2.68)	0.35	1.00	1.11 (0.87–1.42)	0.39	1.02 (0.78–1.34)	0.87	
Type of first-line treatment								0.67
Endocrine monotherapy (n = 1200 patients, 817 events)	1.49 (0.98–2.28)	0.06	1.00	0.97 (0.82–1.14)	0.69	1.03 (0.85–1.23)	0.78	
Endocrine therapy+CDK 4/6 inhibitor (n = 256 patients, 126 events)	1.05 (0.32–3.45)	0.94	1.00	1.31 (0.84–2.04)	0.23	1.19 (0.76–1.86)	0.45	

Analyses adjusted for age, WHO performance status, presence of comorbidities, MFI, number of metastatic sites, and site of metastases. Age was excluded as a confounding factor in the stratified analyses by age

*ABC *advanced breast cancer, *BMI *body mass index, *CDK 4/6 inhibitor *cyclin-dependent kinase 4/6 inhibitor, *CI *confidence interval, *HR *hazard ratio, *HR+/HER2− *hormone receptor-positive/human epidermal growth factor receptor-2-negative, *MFI *metastatic-free interval

^a^The full multivariable model for the total study population is displayed in Supplementary Table 2

### Prognostic impact of BMI on PFS

The PFS was not statistically significantly different between BMI classes, with a median PFS of the first-given systemic therapy of 15.6 months (95% CI 7.6–20.5) in underweight, 15.5 months (95% CI 13.4–16.7) in normal weight, 16.8 months (95% CI 15.2–19.2) in overweight, and 17.3 months (95% CI 14.7–19.1) in obese patients (log-rank p-value = 0.16) (Fig. 2b). After adjustment for potential confounders, when compared with normal weight patients, no statistically significant differences in PFS were observed in underweight (HR 1.05; 95% CI 0.73–1.51; p = 0.81), overweight (HR 0.90; 95% CI 0.79–1.03; p = 0.14), or obese patients (HR 0.88; 95% CI 0.76–1.02; p = 0.10) (Table 3). Similar results were observed in patients with metachronous metastases after additional correction for endocrine sensitivity (Supplementary Figure 2b and Supplementary Table 3; model 3). No signs of effect modification by either age at diagnosis, period of treatment, or type of first-line treatment were present (Table 3).

**Table 3:** Multivariable analyses of first-line progression-free survival according to BMI class at diagnosis of HR+/HER2− ABC in the total study population and subgroups of patients

	BMI							p interaction
	Underweight		Normal weight	Overweight		Obese		
	HR (95% CI)	p-value	Reference	HR (95% CI)	p-value	HR (95% CI)	p-value	
Progression-free survival								
All patients (n = 1456 patients, 1184 events)^a^	1.05 (0.73–1.51)	0.81	1.00	0.90 (0.79–1.03)	0.14	0.88 (0.76–1.02)	0.10	
Age at diagnosis of ABC								0.55
< 60 years (n = 398 patients, 326 events)	0.72 (0.33–1.59)	0.42	1.00	1.02 (0.78–1.32)	0.89	1.00 (0.75–1.33)	0.99	
≥ 60 years (n = 1058 patients, 858 events)	1.10 (0.73–1.68)	0.64	1.00	0.88 (0.75–1.04)	0.13	0.86 (0.72–1.03)	0.10	
Period of treatment								0.52
2007–2011 (n = 179 patients, 169 events)	1.34 (0.58–3.12)	0.49	1.00	0.84 (0.56–1.26)	0.40	1.26 (0.83–1.90)	0.27	
2012–2016 (n = 518 patients, 462 events)	1.17 (0.61–2.23)	0.64	1.00	0.91 (0.74–1.13)	0.39	0.89 (0.70–1.15)	0.38	
2017–2021 (n = 759 patients, 553 events)	0.96 (0.56–1.65)	0.89	1.00	0.89 (0.72–1.08)	0.24	0.79 (0.64–0.98)	0.03	
Type of first-line treatment								0.89
Endocrine monotherapy (n = 1200 patients, 1005 events)	1.09 (0.74–1.62)	0.66	1.00	0.90 (0.78–1.04)	0.17	0.89 (0.76–1.05)	0.18	
Endocrine therapy + CDK4/6 inhibitor (n = 256 patients, 179 events)	0.83 (0.30–2.30)	0.72	1.00	1.00 (0.69–1.43)	0.98	0.85 (0.58–1.23)	0.39	

Analyses adjusted for WHO performance status, presence of comorbidities, MFI, number of metastatic sites, and site of metastases

*ABC *advanced breast cancer, *BMI *body mass index, *CDK 4/6 inhibitor *cyclin-dependent kinase 4/6 inhibitor, *CI *confidence interval, *HR *hazard ratio, *HR+/HER2− *hormone receptor-positive/human epidermal growth factor receptor-2-negative, *MFI *metastatic-free interval

^a^The full multivariable model for the total study population is displayed in Supplementary Table 3

## Discussion

In this study on a real-world cohort of 1456 patients diagnosed with HR+/HER2− ABC who received endocrine therapy with or without a CDK 4/6 inhibitor as first-given systemic therapy in the Netherlands between 2007 and 2020, we evaluated whether BMI is an independent prognostic factor for OS and PFS. In contrast to the findings in patients with EBC, we observed that neither overweight nor obesity was associated with either OS or PFS. Interestingly, however, we observed that underweight patients tended to have a lower OS when compared with normal weight patients.

Our results regarding the lack of association between a higher BMI and breast cancer outcomes are consistent with the results of other studies on patients diagnosed with HR+/HER2− ABC [11–13, 15]. In a recent study of the French ESME cohort, for example, both overweight and obesity did not seem to affect the OS of 7844 patients diagnosed with HR+/HER2− ABC with a HR of 0.95 (95% CI 0.88–1.03) and a HR of 0.99 (95% CI 0.90–1.08), respectively, using normal weight as the reference [12]. Correspondingly, in a large pooled analysis of the MONARCH 2 and 3 trials including 1138 patients diagnosed with HR+/HER2− ABC who received either endocrine monotherapy or endocrine therapy in combination with abemaciclib, no differences in PFS were observed between normal weight and overweight and obese patients in both treatment arms [13]. These results are further corroborated by a study among 219 women with HR+ABC on first- or second-line treatment with an aromatase inhibitor, in which no difference in PFS was observed between patients with a BMI of < 27 kg/m^2^ and patients with a BMI of ≥ 27 kg/m^2^ [15]. Therefore, our results add to the available evidence on the lack of a prognostic effect of overweight and obesity in patients diagnosed with HR+/HER2− ABC.

The lack of a prognostic effect of both overweight and obesity in our cohort of patients with HR+/HER2− ABC stands in strong contrast with the well-documented adverse prognostic effect of overweight and obesity in patients with EBC [5, 6]. For example, in a meta-analysis including patients diagnosed with HR+/HER2− EBC, obesity resulted in a statistically significant decrease in both disease-free survival (DFS) (HR 1.26; 95% CI 1.13–1.41) and OS (HR 1.39; 95% CI 1.20–1.62) when compared with normal weight [5]. The lack of a prognostic effect of overweight and obesity in patients with HR+/HER2− ABC might potentially be explained by the recently emerged “obesity paradox’’. This phenomenon is defined by the finding of an inverse rather than an adverse association between a higher BMI and (breast cancer) outcomes; a finding which has been observed in several studies among patients with metastatic cancer [28–33]. Potential mechanisms for the obesity paradox comprise both methodological and clinical explanations [28, 29]. Methodological explanations, for example, include the use of BMI as an inadequate measurement tool for adiposity, confounding by smoking, detection bias, and reverse causation. On the other hand, clinical explanations include the presence of less aggressive tumours in obese patients, an enhanced treatment response in obese patients, and a greater energy reserve that may confer a survival benefit in the treatment of ABC.

An interesting finding of our study is the adverse prognostic effect associated with an underweight BMI classification, though results were not statistically significant and limited by the small number of underweight patients included in this study. Specifically, we observed that underweight patients tended to have a lower OS when compared with normal weight patients (HR 1.45; 95% CI 0.97–2.15). In the French ESME study mentioned earlier, underweight (versus normal weight) was also identified as a negative prognostic factor for OS (HR 1.11; 95% CI 1.01–1.22) [12]. Moreover, an adverse association between underweight and OS has also been observed in patients with EBC [34, 35]. However, BMI does not distinguish between lean tissue and fat tissue, and may therefore not be the most appropriate measurement tool for body composition, and sarcopenia in particular [36]. It is important to mention this limitation of BMI as several smaller cohort studies have shown that sarcopenia is associated with an adverse prognosis in ABC [37–39]. Hence, the adverse prognostic effect of underweight may also be related to the presence of sarcopenia in our cohort.

The use of a large prospectively maintained retrospective cohort study including all patients diagnosed with HR+/HER2− ABC in the southeast of the Netherlands is a major strength of our study. The classification of underweight patients as a separate group is another strength of our study, even though the small number of patients impacted the power of the results. Our study also has some limitations. We did not collect information about BMI or weight change prior to diagnosis of ABC. It is possible that underweight patients lost weight shortly before diagnosis of ABC as a result of more aggressive disease, and consequently experienced an adverse prognosis. This phenomenon is referred to as ‘reverse causation’. In addition, 764 patients with HR+/HER2− ABC did not have a BMI measurement at diagnosis and were consecutively excluded from this study, possibly introducing selection bias.

In this large prospectively maintained retrospective cohort study including 1456 patients diagnosed with HR+/HER2− ABC, overweight and obesity were prevalent, while underweight was uncommon. In contrast to the findings in EBC, we showed that overweight and obesity do not impact the prognosis of patients with ABC. This lack of association was observed regardless of age at diagnosis of ABC, period of treatment, or type of first-line treatment. Interestingly, at the same time, we showed that underweight is a potential adverse prognostic factor for OS. However, as only a limited number of underweight patients were included in this study and information about BMI before ABC diagnosis and the presence of sarcopenia was lacking, our results should be considered as hypothesis-generating and therefore need to be confirmed in other studies. Nonetheless, these findings stress the importance of recognising underweight patients as a separate group of patients and support adequate monitoring of underweight patients.

### Supplementary Information

Below is the link to the electronic supplementary material.

Supplementary file1 (PDF 783 KB)

## Data Availability

Data will be shared with interested researchers who are able to provide a methodologically sound proposal with well-defined research questions. Researchers are welcome to contact the corresponding author for more information at vcg.tjan.heijnen@mumc.nl.

## Abbreviations

ABC: Advanced breast cancer
BMI: Body mass index
CI: Confidence interval
CDK 4/6: Cyclin-dependent kinase 4/6
DFS: Disease-free survival
EBC: Early breast cancer
HER2: Human epidermal growth factor receptor 2
HR+: Hormone receptor-positive
HR: Hazard ratio
IQR: Interquartile range
MFI: Metastatic-free interval
OS: Overall survival
PFS: Progression-free survival
