# Supplementary material for: The prognostic impact of BMI in patients with HR+/HER2− advanced breast cancer: a study of the SONABRE registry
Source: Breast Cancer Res Treat. 2023 Oct 25;203(2):339–49. doi: 10.1007/s10549-023-07108-6 (PMC10787675; doi:10.1007/s10549-023-07108-6)
Supplement: Supplementary file 1 — Supplementary file1 (PDF 783 KB) [file 10549_2023_7108_MOESM1_ESM.pdf]

Supplementary appendix

Supplement to: Lammers S.W.M., Thurisch H., Vriens I.J.H., et al. The prognostic impact of BMI in patients with HR+/HER2- advanced breast cancer: a study of the SONABRE registry.

Corresponding author:
Vivianne C.G. Tjan-Heijnen, MD, PhD
ORCiD ID: 0000-0002-3935-7440
Department of Medical Oncology, Maastricht University Medical Centre
P.O. Box 5800, 6202 AZ Maastricht, the Netherlands
Email: vcg.tjan.heijnen@mumc.nl
Phone: +31433877025

Table of contents

Supplementary Table 1. Baseline characteristics of patients with metachronous metastases according to BMI class at diagnosis of HR+/HER2- ABC (N (%))............ 3

Supplementary Fig. 1 Type of first-given systemic therapy in patients diagnosed with HR+/HER2- ABC between 2017 and 2020, according to BMI class at diagnosis............ 5

Supplementary Fig. 2 Overall survival (a) and first-line progression-free survival (b) according to BMI class at diagnosis of HR+/HER2- ABC in patients with metachronous metastases 6

Supplementary Table 2. Multivariable analyses of overall survival............ 7

Supplementary Table 3. Multivariable analyses of first-line progression-free survival 9

Supplementary Table 1. Baseline characteristics of patients with metachronous metastases according to BMI class at diagnosis of HR+/HER2- ABC (N (%))

	**Total** n = 1,084 (100%)	**Underweight** n = 20 (2%)	**Normal weight** n = 460 (42%)	**Overweight** n = 359 (33%)	**Obese** n = 245 (23%)	**p-value**
Sex						0.84
Female	1,070 (99)	20 (100)	455 (99)	353 (98)	242 (99)	
Median age – years (range)	68 (29-94)	63 (47-88)	68 (29-94)	68 (36-93)	68 (38-89)	0.67
Age						0.51
<60 years	279 (26)	7 (35)	126 (27)	87 (24)	59 (24)	
≥60 years	805 (74)	13 (65)	334 (73)	272 (76)	186 (76)	
WHO performance status						0.002
0-1	831 (84)	10 (59)	351 (85)	293 (87)	177 (79)	
2-4	160 (16)	7 (41)	63 (15)	43 (13)	47 (21)	
Unknown	93	3	46	23	21	
Comorbidities[a]						<0.001
No	382 (35)	6 (30)	191 (42)	121 (34)	64 (26)	
Yes	702 (65)	14 (70)	269 (59)	238 (66)	181 (74)	
MFI						0.16
3-60 months	354 (33)	5 (25)	138 (30)	118 (33)	93 (38)	
>60 months	730 (67)	15 (75)	322 (70)	241 (67)	152 (62)	
Number of metastatic sites						0.22
Single	497 (46)	10 (50)	195 (42)	169 (47)	123 (50)	
Multiple	587 (54)	10 (50)	265 (58)	190 (53)	122 (50)	
Site of metastases						0.02
Bone only	355 (33)	4 (20)	120 (28)	128 (36)	93 (38)	
Soft tissue, without visceral or CNS manifestations	129 (12)	2 (10)	51 (11)	38 (11)	38 (16)	
Visceral or other, without CNS manifestations	565 (52)	14 (70)	262 (57)	179 (50)	110 (45)	
CNS	35 (3)	0 (0)	17 (4)	14 (4)	4 (2)	
(Neo)adjuvant treatment						0.85
No (incl. MFI < 3 months)	293 (27)	5 (25)	127 (28)	100 (28)	61 (25)	
Yes	791 (73)	15 (75)	333 (72)	259 (72)	184 (75)	
(Neo)adjuvant endocrine treatment						0.52
No (incl. MFI < 3 months)	338 (31)	5 (25)	150 (33)	115 (32)	68 (28)	
Yes	746 (69)	15 (75)	310 (67)	244 (68)	177 (72)	
Endocrine sensitivity						0.04
Resistant	354 (33)	7 (35)	129 (28)	126 (35)	92 (38)	
Sensitive	730 (67)	13 (65)	331 (72)	233 (65)	153 (62)	
Type of first-line endocrine therapy for ABC						0.33
Aromatase inhibitor	842 (78)	17 (85)	353 (77)	287 (80)	185 (76)	
Tamoxifen	124 (11)	3 (15)	59 (13)	37 (10)	25 (10)	
Fulvestrant	115 (11)	0 (0)	47 (10)	35 (10)	33 (14)	
Other	3 (<1)	0 (0)	1 <1)	0 (0)	2 (1)	

Abbreviations: ABC = advanced breast cancer; CNS = central nervous system; HR+/HER2- = hormone receptor-positive/ human epidermal growth factor receptor-2-negative; MFI = metastatic-free interval; WHO = World Health Organization.

Data in each cell represent numbers with associated percentages, unless otherwise specified.
Percentages may exceed 100% due to rounding.

[a]The definition of comorbidities includes the following conditions: diabetes mellitus, cerebrovascular disease, connective tissue disease, dementia, hypertension, chronic pulmonary disease, liver disease, renal disease, AIDS, peptic ulcer, leukaemia, malignant lymphoma, myocardial infarction, malignant solid tumour (excluding breast cancer and basal cell carcinoma of the skin), osteoporosis, chronic bowel disease, chronic infectious disease, atherosclerosis, heart failure, mental health conditions, thromboembolism, and heart rhythm disorders.

Supplementary Fig. 1 Type of first-given systemic therapy in patients diagnosed with HR+/HER2- ABC between 2017 and 2020, according to BMI class at diagnosis

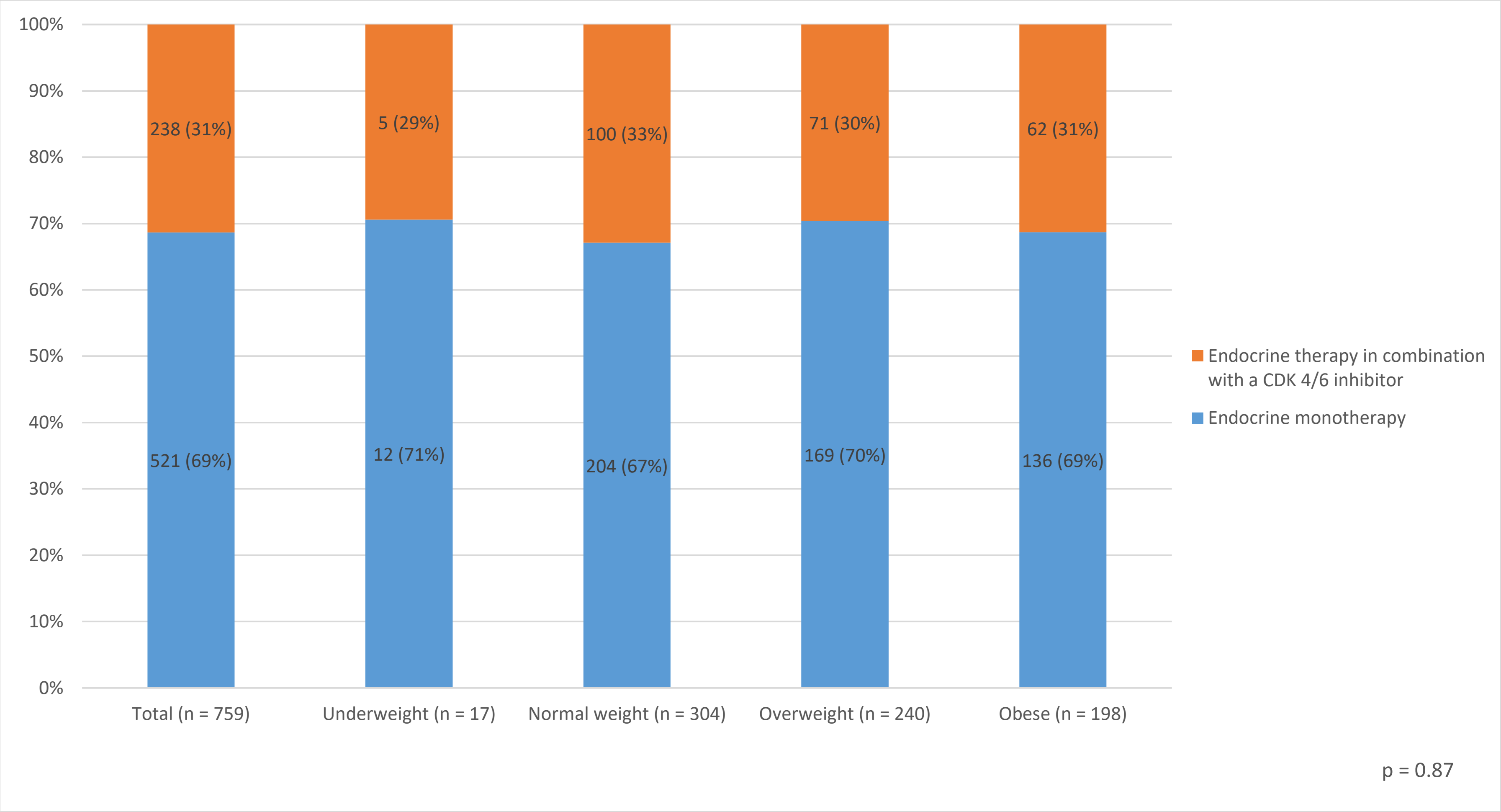

Supplementary Fig. 2 Overall survival (a) and first-line progression-free survival (b) according to BMI class at diagnosis of HR+/HER2- ABC in patients with metachronous metastases

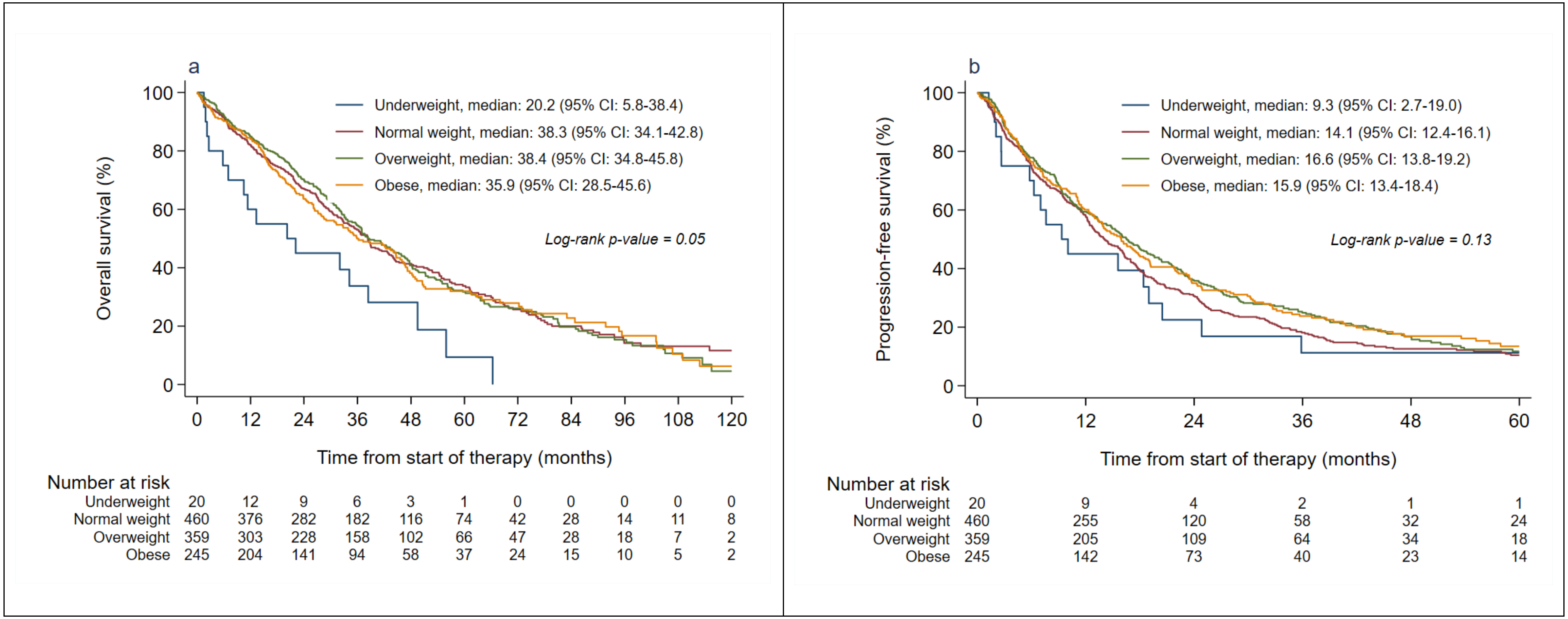

Supplementary Table 2. Multivariable analyses of overall survival

	n (%)	All patients (n = 1,456 patients, 943 events)		n (%)	Patients with metachronous metastases (n = 1,084 patients, 715 events)			
		Model 1			Model 2		Model 3	
		Hazard ratio (95% CI)	p-value		Hazard ratio (95% CI)	p-value	Hazard ratio (95% CI)	p-value
BMI								
Underweight	35 (2)	1.45 (0.97-2.15)	0.07	20 (2)	1.84 (1.12-3.03)	0.02	1.85 (1.13-3.05)	0.02
Normal weight	580 (40)	Ref		460 (42)	Ref		Ref	
Overweight	479 (33)	0.99 (0.85-1.16)	0.93	359 (33)	0.99 (0.83-1.17)	0.90	0.95 (0.80-1.13)	0.57
Obese	362 (25)	1.04 (0.88-1.24)	0.62	245 (23)	1.01 (0.83-1.23)	0.90	0.99 (0.81-1.20)	0.89
Age								
18-55 years	267 (18)	Ref		181 (17)	Ref		Ref	
56-75 years	793 (54)	1.10 (0.91-1.32)	0.32	624 (58)	0.99 (0.80-1.22)	0.90	1.08 (0.87-1.33)	0.50
>75 years	396 (27)	1.39 (1.12-1.73)	0.002	279 (26)	1.14 (0.88-1.47)	0.31	1.29 (1.00-1.66)	0.05
WHO performance status								
0-1	1,103 (76)	Ref		831 (77)	Ref		Ref	
2-4	227 (16)	1.81 (1.51-2.17)	<0.001	160 (15)	1.90 (1.53-2.34)	<0.001	1.88 (1.52-2.32)	<0.001
Unknown	126 (9)	1.29 (1.05-1.58)	0.02	93 (9)	1.28 (1.01-1.62)	0.04	1.36 (1.07-1.72)	0.01
Comorbidities								
No	521 (36)	Ref		382 (35)	Ref		Ref	
Yes	935 (64)	1.26 (1.08-1.46)	0.003	702 (65)	1.28 (1.08-1.52)	0.005	1.30 (1.10-1.54)	0.002
Metastatic-free interval								
<3 months	372 (26)	Ref						
3-60 months	354 (24)	1.64 (1.37-1.97)	<0.001	354 (33)	Ref		Ref	
>60 months	730 (50)	0.92 (0.78-1.09)	0.34	730 (67)	0.57 (0.49-0.67)	<0.001	0.76 (0.64-0.91)	0.003
Number of metastatic sites								
Single	691 (47)	Ref		497 (46)	Ref		Ref	
Multiple	765 (53)	1.58 (1.28-1.96)	<0.001	587 (54)	1.47 (1.16-1.87)	0.002	1.50 (1.18-1.91)	0.001
Site of metastases								
Bone only	507 (35)	Ref		355 (33)	Ref		Ref	
Soft tissue without visceral or CNS manifestations	184 (13)	0.64 (0.48-0.86)	0.003	129 (12)	0.64 (0.45-0.90)	0.01	0.65 (0.46-0.92)	0.01
Visceral or other, without CNS manifestations	725 (50)	0.97 (0.77-1.23)	0.83	565 (52)	1.05 (0.81-1.37)	0.70	1.05 (0.81-1.37)	0.69
CNS	40 (3)	1.59 (1.07-2.36)	0.02	35 (3)	1.45 (0.94-2.23)	0.10	1.35 (0.88-2.07)	0.17
Endocrine sensitivity								

	Sensitive		-		730 (67)	-		Ref	
	Resistant		-		354 (33)	-		1.95 (1.63-2.34)	<0.001

Supplementary Table 3. Multivariable analyses of first-line progression-free survival

	n (%)	All patients (n = 1,456 patients, 1184 events)		n (%)	Patients with metachronous metastases (n = 1,084 patients, 880 events)			
		Model 1			Model 2		Model 3	
		Hazard ratio (95% CI)	p-value		Hazard ratio (95% CI)	p-value	Hazard ratio (95% CI)	p-value
BMI								
Underweight	35 (2)	1.05 (0.73-1.51)	0.81	20 (2)	1.13 (0.70-1.82)	0.61	1.18 (0.73-1.91)	0.49
Normal weight	580 (40)	Ref		460 (42)	Ref		Ref	
Overweight	479 (33)	0.90 (0.79-1.03)	0.14	359 (33)	0.87 (0.75-1.02)	0.09	0.86 (0.74-1.01)	0.06
Obese	362 (25)	0.88 (0.76-1.02)	0.10	245 (23)	0.85 (0.71-1.02)	0.07	0.86 (0.72-1.02)	0.09
WHO performance status								
0-1	1,103 (76)	Ref		831 (77)	Ref		Ref	
2-4	227 (16)	1.38 (1.17-1.63)	<0.001	160 (15)	1.39 (1.14-1.70)	0.001	1.36 (1.11-1.65)	0.002
Unknown	126 (9)	1.23 (1.01-1.50)	0.04	93 (9)	1.25 (1.00-1.56)	0.05	1.24 (0.99-1.55)	0.06
Comorbidities								
No	521 (36)	Ref		382 (35)	Ref		Ref	
Yes	935 (64)	1.10 (0.97-1.24)	0.13	702 (65)	1.09 (0.95-1.26)	0.22	1.12 (0.97-1.30)	0.11
Metastatic-free interval								
<3 months	372 (26)	Ref			-		-	
3-60 months	354 (24)	1.39 (1.19-1.64)	<0.001	354 (33)	Ref		Ref	
>60 months	730 (50)	0.90 (0.78-1.04)	0.14	730 (67)	0.65 (0.57-0.75)	<0.001	0.86 (0.73-1.01)	0.07
Number of metastatic sites								
Single	691 (47)	Ref		497 (46)	Ref		Ref	
Multiple	765 (53)	1.41 (1.17-1.70)	<0.001	587 (54)	1.30 (1.05-1.60)	0.02	1.33 (1.08-1.64)	0.008
Site of metastases								
Bone only	507 (35)	Ref		355 (33)	Ref		Ref	
Soft tissue without visceral or CNS manifestations	184 (13)	0.82 (0.64-1.05)	0.12	129 (12)	0.84 (0.63-1.11)	0.22	0.86 (0.65-1.15)	0.32
Visceral or other, without CNS manifestations	725 (50)	1.06 (0.86-1.29)	0.59	565 (52)	1.12 (0.89-1.41)	0.34	1.14 (0.91-1.44)	0.26
CNS	40 (3)	1.36 (0.93-2.01)	0.12	35 (3)	1.33 (0.87-2.03)	0.20	1.24 (0.81-1.90)	0.32
Endocrine sensitivity								
Sensitive		-		730 (67)	-		Ref	
Resistant		-		354 (33)	-		1.90 (1.62-2.24)	<0.001
